# Predictors and Prevalence of Latent Tuberculosis Infection in Patients Receiving Long-Term Hemodialysis and Peritoneal Dialysis

**DOI:** 10.1371/journal.pone.0042592

**Published:** 2012-08-20

**Authors:** Chin-Chung Shu, Vin-Cent Wu, Feng-Jung Yang, Sung-Ching Pan, Tai-Shuan Lai, Jann-Yuan Wang, Jann-Tay Wang, Li-Na Lee

**Affiliations:** 1 Department of Traumatology, National Taiwan University Hospital, Taipei City, Taiwan; 2 College of Internal Medicine, National Taiwan University, Taipei City, Taiwan; 3 Department of Internal Medicine, National Taiwan University Hospital, Taipei City, Taiwan; 4 Department of Internal Medicine, National Taiwan University Hospital, Yun-Lin Branch, Yun-Lin County, Taiwan; 5 Department of Internal Medicine, National Taiwan University Hospital, Bei-Hu Branch, Taipei City, Taiwan; 6 Department of Laboratory Medicine, National Taiwan University Hospital, Taipei City, Taiwan; University of Sao Paulo Medical School, Brazil

## Abstract

**Background:**

Tuberculosis is a common infectious disease in long-term dialysis patients. The prevalence of latent tuberculosis infection (LTBI) in this population is unclear, particularly in those receiving peritoneal dialysis (PD). This study investigated the prevalence of LTBI in patients receiving either hemodialysis (HD) or PD to determine predictors of LTBI and indeterminate results of interferon-gamma release assay.

**Methods:**

Patients receiving long-term (≥3 months) HD or PD from March 2011 to February 2012 in two medical centers were prospectively enrolled. QuantiFERON-Gold in tube (QFT) test was used to determine the status of LTBI after excluding active tuberculosis. The LTBI prevalence was determined in patients receiving different dialysis modes to obtain predictors of LTBI and QFT-indeterminate results.

**Results:**

Of 427 patients enrolled (124 PD and 303 HD), 91 (21.3%) were QFT-positive, 316 (74.0%) QFT-negative, and 20 (4.7%) QFT-indeterminate. The prevalence of LTBI was similar in the PD and HD groups. Independent predictors of LTBI were old age (OR: 1.034 [1.013–1.056] per year increment), TB history (OR: 6.467 [1.985–21.066]), and current smoker (OR: 2.675 [1.061–6.747]). Factors associated with indeterminate QFT results were HD (OR: 10.535 [1.336–83.093]), dialysis duration (OR: 1.113 [1.015–1.221] per year increment), anemia (OR: 8.760 [1.014–75.651]), and serum albumin level (OR: 0.244 [0.086–0.693] per 1 g/dL increment).

**Conclusion:**

More than one-fifth of dialysis patients have LTBI. The LTBI prevalence is similar in PD and HD patients but is higher in the elderly, current smokers, and those with prior TB history. Such patients require closer follow-up. Repeated or alternative test may be required for malnutrition patients who received long length of HD.

## Introduction

Tuberculosis (TB) remains a worldwide infectious disease with high mortality. Control involves preventing further TB transmission via early diagnosis and treatment of latent TB infection (LTBI) [1]. Dialysis patients are at increased risk of tuberculosis (TB) due to attenuated cellular immunity [2]. Compared to the general population, their risk of developing active TB is 7.8–25 times higher [3]–[5] and their mortality rate due to TB is also higher [4]–[7]. Moreover, TB diagnosis in dialysis patients is usually delayed because of frequent extra-pulmonary manifestations [8], [9]. Early LTBI detection and monitoring of the development of active TB in this specific group are therefore important [1]. Currently, interferon-gamma release assays (IGRAs) used to determine LTBI cases, though not 100% accurate, have been proven useful even in immuno-compromised hosts and Bacille Calmette Guérin (BCG)-vaccinated subjects [10]–[12]. For patients receiving hemodialysis, the IGRA-positive rate is reportedly around 21–40% [13]–[15], while 6–11% of patients have indeterminate IGRA results [14]–[17]. However, studies focusing on patients receiving peritoneal dialysis (PD) are lacking.

Traditionally, HD patients are believed to have higher prevalence of LTBI than PD patients due to more frequent hospital visits and longer hospital stay [14]. In fact, studies supporting this argument are lacking. Understanding the predictors for LTBI and indeterminate IGRA results in the dialysis population is important in policy-making to determine the priority groups for IGRA screening [18], [19]. This cross sectional study was conducted to analyze the prevalence of LTBI in patients receiving long-term HD and PD, and to examine the predictors for LTBI and indeterminate IGRA results.

## Methods

This cross sectional study was conducted at National Taiwan University Hospital, a tertiary referral center in northern Taiwan, and its branch in southern Taiwan. The hospital's institutional review board approved the study. From March 2011 to February 2012, adult patients (age ≥20 years) receiving long-term (>3 months) dialysis were prospectively identified. All participants provided written informed consent. Chest radiography and clinical history were obtained to exclude active TB disease. Acid-fast smear and mycobacterial culture for three sputum samples were performed as previously described if TB was suspected [20]. Those with human immunodeficiency virus infection, liver cirrhosis of Child-Pugh class C [21], cancer or autoimmune disease receiving chemotherapy within the last three months, life expectancy of less than 6 months, and active TB within the last three years were excluded.

Peripheral blood samples were taken to detect LTBI using QuantiFERON-TB Gold In-Tube assay (QFT) (Celestis, Australia), which was performed according to the manufacturer's instructions [22]. Interferon-gamma level of the post-reaction supernatant was then measured by enzyme-linked immuno-sorbent assay (ELISA) and results were interpreted as positive, negative, or indeterminate accordingly [23], [24]. In this study, LTBI was defined as a positive QFT result.

### Data collection

Demographic and clinical data, including age, sex, underlying co-morbidities, prior TB history, contact history of TB, respiratory and constitutional symptoms, smoking status, and blood hemoglobin and serum albumin levels were recorded in a standardized case report form. Dialysis mode was defined as its use in the past three months prior to the QFT test. Every session of HD regularly lasted for 4 hours according to the National Kidney Foundation Kidney Disease Outcome Quality Initiative (NKF KDOQI) [25], with two-to-three sessions per week depending on the patient's residual renal function and the adequacy of dialysis. Peritoneal dialysis was recorded as continuous ambulatory peritoneal dialysis (CAPD) or automated peritoneal dialysis (APD). Hypoalbuminemia was defined as serum albumin level <3.5 g/dL [26] and anemia as hemoglobin level <12 g/dL in males and <11 g/dL in females. Cough ≥3 weeks was defined as chronic cough. Current smoker was defined as those who had smoked >100 cigarettes, with the latest time of smoking within one month prior to the study [27].

Chest radiography findings were classified into “no lung parenchymal lesion”; “lung lesion not compatible with TB”; or “lung lesion compatible with prior TB”. The lung lesion compatible with TB was defined as new patch(es) of consolidation, collapse, lymphadenopathy, mass or nodule, or cavitary lesion without other proven etiology [5]. Prior TB was defined radiographically as fibrotic infiltrates with pleural thickening or calcified nodules over the upper lung fields and other fibrotic lesions documented from previous TB disease [28].

### Statistical analysis

Subjects were classified according to LTBI status for further comparison. Inter-group differences were analyzed using the student *t* test for numerical variables and *chi*-square test for categorical variables. Multivariate logistic regression analysis was used to identify factors associated with LTBI and QFT-indeterminate results. All potential predictors were included in the stepwise variable selection procedure. A two-sided *p*<0.05 was considered significant. All analyses were performed using the SPSS (Version 13.0, Chicago, IL).

## Results

A total of 427 subjects (mean age, 61.1±13.1 years; male, 53%) with long-term dialysis (mean length of dialysis use, 4.8±4.2 years) were enrolled, including 303 HD patients and 124 PD patients (Table 1). Among HD patients, 271 (89.4%) had three sessions per week while the remaining 32 (10.6%) had two sessions per week. Among PD patients, 81 (65.3%) had CAPD and 43 (34.7%) had APD.

**Table 1:** Clinical characteristics of patients with different modes of dialysis.

	PD group (n = 124)	HD group (n = 303)	*p* value
Age, year	55.4±12.3	63.5±12.7	<0.001
Male gender	54 (44%)	172 (57%)	0.013
Smoking status			
Ex-smoker	17 (14%)	65 (22%)	0.065
Current smoker	5 (4%)	22 (7%)	0.213
Dialysis duration, years	4.1±3.9	5.0±4.3	0.039
Underlying co-morbidity			
Malignancy	5 (4%)	25 (8%)	0.111
Diabetes mellitus	23 (19%)	85 (28%)	0.040
Cirrhosis of liver	3 (2%)	3 (1%)	0.255
Autoimmune disease	4 (3%)	7 (2%)	0.588
Prior TB history	1 (1%)	13 (4%)	0.066
TB exposure history	16 (13%)	23 (8%)	0.084
Symptoms at visit			
Chronic cough with sputum	36 (29%)	27 (9%)	<0.001
Dyspnea	1 (1%)	3 (1%)	0.858
Constitutional symptoms	1 (1%)	1 (1%)	0.513
Chest radiograph around visit			
Lesion, not compatible with TB	22 (18%)	110 (36%)	<0.001
Lesion, compatible with prior TB	0	3 (1%)	0.252
Anemia	99 (79%)	223 (74%)	0.174
Serum albumin, g/dL	3.9±0.4	4.1±0.4	0.002
QFT status			
Positive	24 (19%)	67 (22%)	0.528
Negative	98 (79%)	218 (72%)	0.130
Indeterminate	2 (2%)	18 (6%)	0.055

Abbreviations: PD, peritoneal dialysis; HD, hemodialysis; TB, tuberculosis; QFT, QuantiFERON-TB test.

Data are no. (%) or mean ± standard deviation.

Compared to HD patients, PD patients were significantly younger, predominantly female, more symptomatic with chronic cough, had shorter length of dialysis duration, had lower serum albumin level, and were less likely to have pulmonary lesions on chest radiograph (18% vs. 37%; *p*<0.001). The proportion of QFT-positive patients was similar in the two groups (19% vs. 22%, *p* = 0.109).

The QFT result was positive in 91 (21.3%) patients, negative in 316 (74.0%), and indeterminate in the remaining 20 (4.7%) (Table 2). Among the 20 QFT-indeterminate results, 19 were due to weak response to mitogen and one due to strong response of negative control. The QFT tests were repeated in 14 of the 20 patients and were negative in 9, positive in 1, and indeterminate in the remaining 4.

**Table 2:** Clinical characteristics of patients with different QuantiFERON-TB test (QFT) results.

	QFT-positive (n = 91)	QFT-negative (n = 316)	QFT-indeterminate (n = 20)
Age, year	64.9±11.0*	60.0±13.5	66.2±11.0#
Male gender	55 (60%)	162 (51%)	9 (45%)
Smoking status			
Ex-smoker	22 (24%)	60 (19%)	0#
Current smoker	8 (9%)	16 (5%)	3 (15%)
Dialysis duration: year	4.3±3.6	4.7±4.3	7.5±5.2#
Underlying co-morbidity			
Malignancy	10 (11%)	19 (6%)	1 (5%)
Diabetes mellitus	24 (26%)	78 (25%)	6 (30%)
Cirrhosis of liver	1 (1%)	5 (2%)	0
Autoimmune disease	2 (2%)	9 (3%)	0
Prior TB history	8 (9%)*	6 (2%)	0
TB exposure history	11 (12%)	27 (9%)	1 (5%)
Symptoms at visit			
Chronic cough with sputum	14 (15%)	47 (15%)	2 (10%)
Dyspnea	1 (1%)	1 (1%)	2 (10%)#
Constitutional symptoms	1 (1%)	0	1 (5%)#
Chest radiograph around visit			
Lesion, not compatible with TB	29 (33%)	92 (31%)	11 (55%)#
Lesion, compatible with prior TB	0	2 (1%)	1 (5%)#
Anemia	64 (70%)	240 (76%)	18 (90%)
Serum albumin, g/dL	4.1±0.3	4.0±0.4	3.7±0.4#
HD patients	67 (74%)	218 (69%)	18 (90%)

Abbreviations: HD, hemodialysis; PD, peritoneal dialysis; TB, tuberculosis.

Data are no. (%) or mean ± standard deviation.

* Significant difference (*p*<0.05) between QFT-positive and QFT-negative groups.

# Significant difference (*p*<0.05) between QFT-indeterminate and the combined QFT-negative and QFT-positive groups.

Compared to combined group of QFT- positive and QFT-negative patients, the 20 QFT-indeterminate patients were older and less likely to be ex-smokers, received HD for a longer period, and had more dyspnea and constitutional symptoms (Table 2). They also had lower serum albumin level and a higher proportion of patients with pulmonary lesions. Multivariate logistic regression analysis revealed that dialysis duration (Odds Ratio [O.R.] 1.113, 95% C.I. 1.015–1.221 per year increment), HD (O.R. 10.535, 95% C.I. 1.336–83.093), anemia (O.R. 8.760, 95% C.I. 1.014–75.651), and serum albumin level (O.R. 0.244, 95% C.I. 0.086–0.693 per unit increment) were significant predictors of QFT-indeterminate results (Table 3). If 3.5 g/dl was used as cut-off value for serum albumin level to predict an indeterminate QFT result, sensitivity and specificity were 80% and 93%, respectively.

**Table 3:** Multivariate logistic regression for indeterminate results of QuantiFERON-TB test.

Characteristics	Multivariate	
	*p* value	OR (95% C.I.)
Age, per 1 year increment	0.205	
Gender, male vs. female	0.075	
Smoking, current vs. none	0.130	
Dialysis duration, per 1 year increment	0.023	1.113 (1.015–1.221)
Prior TB history	0.424	
Dialysis mode, HD vs. PD	0.025	10.535 (1.336–83.093)
Anemia, presence vs. absence	0.049	8.760 (1.014–75.651)
Serum albumin, per 1 g/dL increment	0.008	0.244 (0.086–0.693)
Symptoms at examination*	0.052	
Any lesion by chest radiograph	0.135	
Diabetes mellitus	0.388	
Malignancy	0.635	

* Presence of chronic cough, dyspnea, or constitutional symptoms.

**Figure 1:**
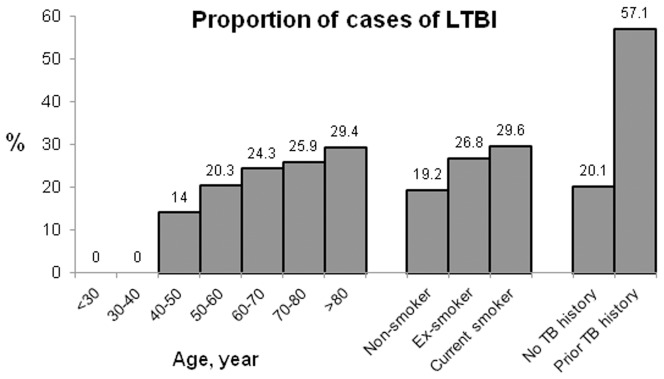
The proportion of latent tuberculosis infection (LTBI) cases defined by QuantiFERON-TB test was plotted according to age, smoking status, and history of TB.

Patients with LTBI (QFT-positive) were older and more likely had a prior TB history compared to QFT-negative patients, by univariate analysis (Table 2). In multivariate logistic regression analysis, independent predictors of LTBI included age (O.R. 1.034, 95% C.I. 1.013–1.056, per year increment), prior TB history (O.R. 6.467, 95% C.I. 1.985–21.066), and current smoker (O.R. 6.467, 95% C.I. 1.985–21.066) (Table 4; Figure 1). The presence of underlying co-morbidity was not an independent predictor. In patients with prior TB history and those who were current smokers, the prevalence of LTBI was 57.1% and 29.6%, respectively. This prevalence increased by <15% in patients younger than 50 years old to almost 30% in those older than 80 years old.

**Table 4:** Multivariate logistic regression for latent tuberculosis (TB) infection diagnosed by QuantiFERON-TB test.

Characteristics	Multivariate	
	*p* value	OR (95% C.I.)
Age, per 1 year increment	0.001	1.034 (1.013–1.056)
Gender, male vs. female	0.677	
Smoking status, current vs. none	0.037	2.675 (1.061–6.747)
Dialysis duration, per 1 year increment	0.142	
Prior TB history, presence vs. absence	0.002	6.467 (1.985–21.066)
Serum albumin, per 1 g/dL increment	0.077	
Symptoms at examination*	0.769	
Any lesion by chest radiograph	0.225	
Diabetes mellitus	0.894	
Malignancy	0.228	

* Presence of chronic cough, dyspnea, or constitutional symptoms.

## Discussion

This is the first cross sectional study that investigated the prevalence of LTBI in a large number of patients receiving either long-term PD or HD. Using QFT as a diagnostic tool for LTBI in this immuno-compromised population, 4.7% may have an indeterminate result, especially those with anemia, hypoalbuminemia, and who have been receiving HD for a long time. There is a high prevalence (21.3%) of LTBI in the long-term dialysis population, especially in the elderly, current smokers, and those with prior TB history. However, the use of either PD or HD is associated with similar risks of LTBI.

Though IGRA-positive is not 100% equivalent of LTBI, it has several advantages over tuberculin skin test (TST) in terms of convenience and accuracy [10]–[12]. The TST has a significant limitation in Taiwan due to BCG vaccination [29] and the high prevalence of NTM disease [30]. In QFT, by incorporating positive control (mitogen) and negative control (no antigen) tubes, the true immune reaction against *Mycobacterium tuberculosis*-specific antigens can be differentiated from false-positive result due to non-specific activation and false-negative result due to immuno-suppression. Thus, IGRA is a better screening test for LTBI than TST while implementing public health policy, especially for an immune-compromised host.

Previous studies using IGRA report an LTBI prevalence of 21–40% in HD patients [13]–[15]. The QFT-positive rate in the present study is within this range and lower than 40%, as reported in a study conducted in south Taiwan [14]. This is probably because the incidence of TB has been decreasing in Taiwan [31]. However, the prevalence in the present study (21.3%) is similar to that reported in household contacts in large-scale studies (11∼30%) [12], [32] and much higher than the results of new health-care staff in the study institute at the same period (13 [5.7%] QFT-positive in 229 [unpublished data]). As such, it can be posited that dialysis patients in Taiwan have a much higher prevalence of LTBI than the general population and should be a priority group for targeted screening for active TB disease, especially IGRA-positive patients. If IGRA is unavailable, focus should be on older patients, current smokers, and those with prior TB history. Quite interestingly, the three predictors for LTBI identified in the present study are also risk factors of active TB disease [14], [33], [34]. Different combinations of these predictors may be useful to select the target population for preventive therapy for LTBI. However, the cost and benefit of preventive therapy in this special population should be further evaluated.

The population of chronic renal failure patients receiving long-term dialysis is increasing worldwide and TB is a commonly associated infectious disease [18], [19]. It has been previously assumed that because HD patients frequently visit the HD room, they are more likely to acquire *Mycobacterium tuberculosis* infection than PD patients via airborne transmission. Only a report of PD patients in Spain has shown a comparable LTBI prevalence of 18% [35]. By simultaneously enrolling HD and PD patients, this is the first study to demonstrate similar LTBI prevalence in the two patient groups, thereby challenging the hypothesis of occult transmission in the HD room. Although the two dialysis groups are different in more ways than just dialysis place and duration, this observation suggests that transmission of TB to HD patients within crowded dialysis facilities may be similar to PD patients at home [36]. This suggests that the study institute has an effective TB infection control policy on early detection, prompt treatment, and rapid isolation.

Although 71.4% of QFT-indeterminate patients have definite results after repeat testing, 4.7% of the initial QFT tests with an indeterminate result are associated with hypoalbuminemia, anemia, HD, and longer dialysis duration. The association between anemia and indeterminate status has been shown before [37]. Along with hypoalbuminemia, these predictors suggest that malnutrition attenuates immune response and compromises the performance of IGRA [38]. The current finding that HD, but not PD, is associated with QFT-indeterminate is interesting and worth discussing further. A previous study reveals that while both HD and PD patients have lower but insignificant HLD-DR expression on peripheral blood monocytes compared to healthy controls, HLA-DR expression is significantly higher in PD than in HD patients [39]. This implies that continuous dialysis like PD can attenuate immune dysfunction compared to intermittent modes like HD. Moreover, longer duration of dialysis in dialysis patients has been correlated with worse cellular immunity [40]. This may explain how a much higher percentage (95%) of QFT-indeterminate results in the present study may come from low mitogen responses, compared to 51% in a public health clinic setting [24]. For diagnosing LTBI in such patients, IGRA should be meticulously applied. Repeating IGRA or using alternative tests may be necessary.

In contrast to a previous report [24], female gender is not an independent factor of QFT-indeterminate results in the present study. This may be due to different patient characteristics, such as age, race, and prevalence of HIV infection, between studies. Further large-scale investigations are necessary to confirm this finding and investigate possible reasons.

The present study has several limitations. First, this study was conducted in a tertiary referral center and its branch, so patients had more underlying co-morbidities and the LTBI prevalence might be higher. Second, without detailed contact investigation, the epidemiologic link and biological implication of QFT-positivity cannot be confirmed. Lastly, this is a cross-sectional study. Further prospective studies with long-term follow-up on the development of active TB are needed.

In conclusion, patients receiving PD have a similar prevalence of LTBI as those receiving HD (19% and 22%, respectively). The prevalence of LTBI in long-term dialysis patients is even higher in the elderly, current smokers, and those with prior TB history. These risk factors can be used to select a target group for cost-effective LTBI screening. Patients receiving HD or long duration of dialysis, and those with anemia or lower albumin level are likely to have a QFT-indeterminate result. For such patients, repeat IGRA or alternative test may be necessary to detect LTBI.

### Disclosures

Parts of the study results have been presented as a poster in the 2011 Congress of the Asia Pacific Society of Respirology and the 2012 International Conference of the American Thoracic Society.
